# Silkworm Sericin: Properties and Biomedical Applications

**DOI:** 10.1155/2016/8175701

**Published:** 2016-11-14

**Authors:** Regina Inês Kunz, Rose Meire Costa Brancalhão, Lucinéia de Fátima Chasko Ribeiro, Maria Raquel Marçal Natali

**Affiliations:** ^1^Department of Morphological Sciences, State University of Maringá, Av. Colombo, 5790, 87020-900 Maringá, PR, Brazil; ^2^Center of Biological Sciences and Health, State University of Western Paraná, Rua Universitária, 2069, 85819-110 Cascavel, PR, Brazil

## Abstract

Silk sericin is a natural polymer produced by silkworm,* Bombyx mori*, which surrounds and keeps together two fibroin filaments in silk thread used in the cocoon. The recovery and reuse of sericin usually discarded by the textile industry not only minimizes environmental issues but also has a high scientific and commercial value. The physicochemical properties of the molecule are responsible for numerous applications in biomedicine and are influenced by the extraction method and silkworm lineage, which can lead to variations in molecular weight and amino acid concentration of sericin. The presence of highly hydrophobic amino acids and its antioxidant potential make it possible for sericin to be applied in the food and cosmetic industry. The moisturizing power allows indications as a therapeutic agent for wound healing, stimulating cell proliferation, protection against ultraviolet radiation, and formulating creams and shampoos. The antioxidant activity associated with low digestibility of sericin that expands the application in the medical field, such as antitumour, antimicrobial and anti-inflammatory agent, anticoagulant, acts in colon health, improving constipation and protects the body from obesity through improved plasma lipid profile. In addition, the properties of sericin allow its application as a culture medium and cryopreservation, in tissue engineering and for drug delivery, demonstrating its effective use, as an important biomaterial.

## 1. Introduction

Sericin is a protein produced by the silkworm,* Bombyx mori*, a holometabolous insect belonging to the Lepidoptera order and Bombycidae family.* B. mori*, which produces a great amount of sericin to the end of fifth larval instar and together with the fibroin, form the silk thread used in the production of the cocoon, structure that provides the ideal conditions for the occurrence of larval metamorphosis to adults [1].

In the textile industry, the cocoon is processed and sericin is largely removed in a process called degumming. The fibroin is converted into raw silk and used in the production of many types of yarns and silk fabrics [1–3]. The commercial silk farming is considered one of the oldest agricultural explorations made by humans [4]. Historical data is derived from North China, going back to 5000 years. [5], where it has expanded to other areas in East and West.

In addition to its economic importance arising from applications in agribusiness,* B. mori* is the main lepidopteran used in scientific research, a genetic resource capable of elucidating a wide range of biological problems [2]. Recently, the cocoon of the* B. mori* and its main proteins, fibroin and sericin, have been the subject of research that has shown the potential use in the field of polymers, biomaterials, cosmetics, and food industry [3, 6].

For a long time, sericin has been disregarded in the field of sericulture. It is estimated that it 400,000 tons of dry cocoons worldwide, producing 50,000 tons of sericin [7]; sericin is mostly discarded in wastewater. This generates a high chemical and biological oxygen demand as well as contamination of water [8]. The removal and use of sericin could have a strong economic, social, and environmental impact, especially in countries where sericulture is practiced, such as China, India, and Brazil.

The sericin is a natural polymer, which acts as an adhesive joining two fibroin filaments in order to form silk yarn [2]. The molecule is highly hydrophilic with a molecular weight that ranges from 20 to 400 kDa and consists of 18 amino acids, including essentials. The polar groups (carboxyl, hydroxyl, and amino groups) of amino acid side chains and its organic composition, solubility, and structural organization enable crosslinking, copolymerizations, and combinations with other polymers, which together convey unique properties to sericin as an antioxidant, moisturizing, healing, antibacterial, antimicrobial protection against ultraviolet radiation, and antitumour [9–11].

Thus, the demand for biocompatible and biodegradable materials shows the growing interest in nontextile applications of silk protein in a vast field in science and medicine and justifies the present review, which has properties and biological applications of sericin polymer obtained from cocoons of* B. mori*.

## 2. Sericin Synthesis and Silk Gland Morphology

The sericin is synthesized in the labial gland of* B. mori*, commonly called silk glands, a pair tubular organ extending lateroventrally to the digestive tract, beginning in the labial segment to the caudal region. In* B. mori*, the gland is rudimentary from 1st to 4th instar producing small amount of silk, which is secreted at the end of each instar and is used to fix the tegument to be discarded after moulting to the substrate. At 5th instar, the gland hypertrophy occurs, an increase in cell volume, high silk biosynthesis, and secretion, and weight estimated to be between 20% and 40% of the total weight of the insect [12–14].

**Figure 1:**
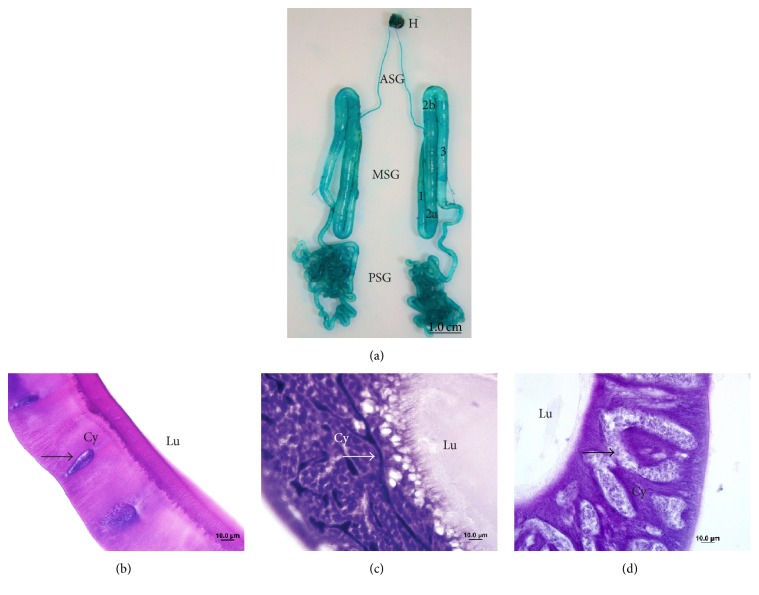
Photography of* Bombyx mori* silk gland (a), stained with light green 1%. Anterior (ASG), middle (MSG), and posterior silk gland (PSG). MSG and its areas: anterior (1), anterior-middle (2a), posterior-middle (2b), and posterior (3). Head (H). In (b), (c), and (d), photomicrographs of different regions anterior, middle, and posterior, respectively, stained with haematoxylin and eosin. Cytoplasm (Cy), nucleus (arrows), and lumen (Lu).

The silk gland is a typical exocrine gland, and, due to morphological and functional differences along the length, it is divided into three regions: the anterior silk gland (ASG) that forms the excretory duct and has about 200 cells; the middle silk gland (MSG) which secretes three types of sericin and is about 7 cm in length and approximately 300 cells; and the posterior silk gland (PSG), secretory of fibroin, that is about 15 cm long and about 500 secreting cells [2, 14–16]. In Figure 1, the pair of silk glands can be visualized, each with its distinct regions. The anterior region ends in a single structure near the head, the silk spinning organ, the spinnerets. The middle region may be subdivided into four areas: anterior, anterior-middle, posterior-middle, and posterior. These areas differ due to the density and morphology of the material contained in secretory vesicles, and each of them synthesizes a different type of sericin in the lumen [16].

The silk gland growth occurs during embryonic development and remains in the larval stage, during which cellular DNA replication occurs without mitosis process known as endomitosis [14].

The glandular epithelium, which resembles morphologically the posterior and middle regions, is simple and relies on a continuous basal lamina that separates the hemocoel. The ultrastructure of the secretory cells reveals numerous nucleoli distributed in the ramified nucleus [16], which contains the large amount of replicated genomic DNA, which increases 200–400 thousand times [17]. At the beginning of larval development, the core surface is regular, becoming convoluted in the course of development [18]. According to Ichimura et al. [19], nuclear ramification starts to be detected at the end of third instar. The cytoplasm is rich in organelles involved in the synthesis of secreted proteins, such as rough endoplasmic reticulum (rough ER), Golgi complex, and mitochondria [16].

Due to the specificities of the middle region of the silk gland, the posterior area secretes the inner layer of sericin, which immediately accumulates around the fibroin. Surrounding the inner layer is deposited a layer of more granular texture, less dense, for the posterior-middle area. The outermost layer of sericin is synthesized by the previous field, which contains the largest cells characterized by the presence of fat bodies [16].

Thus, in the silk thread, the sericin forms three layers around two fibroin filaments coming from each of the silk glands [12]. The mechanism by which soluble silk proteins form an insoluble silk thread is reviewed by Liu and Zhang [20]. The fibroin is secreted into the glandular lumen in solution form with approximately 15% of protein, migrating to the middle region where it is surrounded by the sericin. Gradually, water is absorbed by the cells of the glandular epithelium, forming a solution similar to a gel with 30% of protein, which exhibits the property of nematic liquid crystal. While spinning, the proteins flow through the anterior silk gland duct, where excess water and ions are absorbed, and the crystalline liquid solution solidifies gradually converting into a solid filament. Furthermore, a specific and continuous movement of* B. mori* head during spinning also acts on the orientation of protein molecules in the silk thread, and as the silk proteins aggregate and crystallize, they become more hydrophobic, inducing the loss of water on the surface of the thread [20].

The production of cocoon lasts about three days and occurs from outside in, where silk threads are “glued” using the glue property of the sericin. Cocoon is formed by a long silk thread, whose size ranges from 900 to 1500 meters and its evolution over millions of years provides optimum protection during metamorphosis to silk moth against adverse environmental conditions and attacks of biological agents such as birds, insects, and bacteria. Its main proteins, fibroin and sericin, make up 98% of the structure, besides the presence of the p25 protein and seroin which are probably responsible for the resistance to predators, fungi, and microorganisms, which are also secreted by the silk glands. Other substances such as fats and waxes (0.4 to 0.8%), inorganic salts (0.7%), and pigment (0.2%) are also present in the cocoon [2, 12, 13, 21].

Silk gland undergoes morphological and functional changes resulting from insect metamorphosis, degenerating completely 48 hours after the beginning of the pupal stage [12, 22].

### 2.1. Silk Proteins: Structure and Genetics

Sericin and fibroin are two distinct families of proteins. Fibroin fibre is a glycoprotein secreted into the lumen of the posterior glands as a molecular complex comprising a heavy (H) chain of approximately 350 kDa and a light chain (L) of 25 kDa and P25 with 27 kDa. The L and H genes are located on chromosomes 14 and 25, respectively [23]. Fibroin is arranged as microfibrils organized into fibrils bundles, which together form a single silk filament. During cocooning the two filaments, each filament originating from a silk gland is surrounded by sericin layers and form the silk thread [24].

Sericin is a family of glycoproteins generated by alternative splicing of sericin genes [12, 25], and comprises 25 to 30% of the cocoon weight. The genes expressions are regulated temporally according larval development, which added a little homogeneity between the exons, and it is responsible for the large protein variety [1, 26].

At least three genes are responsible for sericin synthesis:* Ser1*,* Ser2* and* Ser3*. The first discovered gene was* Ser1*, localized in chromosome 11, locus* Src*, that consists of a single copy with about 23 kb and 9 exons and encodes four major mRNAs (10.5, 9.0, 4.0, and 2.8 kb) by alternative splicing [27–30]. Michaille et al. [31] discovered the* Ser2* gene, which contained 13 exons that ranged in size from 28 to 2574 bp and encoded two mRNAs (3.1 and 5.0–6.4 kb) also by alternative splicing.* Ser2* gene proved to be more complex and variable than any other known gene encoding silk proteins. Its gene organization resembled that of the* Ser1* gene, especially the similar size of the first two exons encoding the signal peptides [32]. The last gene involved in sericin synthesis,* Ser3 *gene, was discovered by Takasu et al. [30], and it also is located in chromosome 11, locus* Src-2*. This gene with size about 3.5 bp contains 3 exons and encodes a simple transcript of 4.5 kb [28, 30].

## 3. Sericin Biochemistry

Sericin belongs to a family of proteins of the type “gluelike” which is positioned around the protein core, keeping the fibroin filaments together [4]. In the presence of sericin, fibre silk is hard and tough and becomes soft and lustrous when absent [24].

Structurally, sericin is a globular protein consisting of random coil and *β*-sheets. Changes in random coil structure for *β*-sheet easily occur in response to mechanical stretching properties, moisture absorption, and temperature, where the sol-gel transition occurs. In hot water, 50–60°C or higher, protein adopts its soluble form. At lower temperatures, the solubility is reduced and the random coil structure is converted into *β*-sheets, resulting in the formation of a gel [33–35].

Macromolecule of hydrophilic character is composed of 18 amino acids with strong polar groups such as hydroxyl, carboxyl, and amino groups [36], capable of forming crosslinks, copolymerizations, and combinations with other polymers [37]. Its organic composition is given by 46.5% carbon, 31% oxygen, 16.5% nitrogen, and 6% hydrogen [38]. The biochemical characteristics give sericin important biological properties such as biocompatibility, antibacterial activity, antioxidant, and moisturizing, among others [39].

### 3.1. Forms of Sericin

The solubility and the molecular weight of sericin are properties that can be used as a classification standard. Shaw and Smith [40] classified the sericin in three fractions (A, B, and C) according to its solubility in water. Sericin A, the more soluble fraction in warm water, is found in the outermost layer of the cocoon and contains approximately 17.2% nitrogen, with serine, threonine, glycine, and aspartic acid as major amino acids. In the intermediate layer is found sericin B, which contains 16.8% nitrogen and an addition of tryptophan; it is composed of the same amino acids as sericin A. The last fraction, sericin C, is adjacent to fibroin and it is found in the innermost layer; it is insoluble in hot water and contains a lower proportion of nitrogen, 16.6%. In addition to the amino acids found in sericins A and B, fraction C also contains proline.

Considering the molecular weight, Takasu et al. [41] classified the sericin based in place of synthesis in the middle region of the silk gland, as sericins A, M, and P, that comprises the three largest polypeptides that make up the protein. Sericins P and M are encoded by* Ser1* gene and form the first and second sericin layers that involve the fibroin, respectively [42]. Your transcripts are expressed in the posterior and middle areas of MSG until day 6 from the 5th instar and are not expressed in the anterior area.* Ser2* gene expression is detected in the anterior area; it is rare in the middle area and not expressed in the posterior area. Your expression is detected until day 4 and disappeared after day 6 from fifth instar [43].* Ser3* gene encodes sericin A that occurs mainly in the floss and outer layer of the cocoon [30] and is mostly detected in the anterior area and rare in the middle subpart. The signal of* Ser3* transcript initiates on day 5 and increases in intensity until day 7 from 5th instar [43].

While products of* Ser1* and* Ser3* genes compose the sericin layers in* B. mori* cocoon, the proteins encoded by* Ser2* gene are classified as noncocoon and are related with larval silk [43]. A small amount of silk is spun by silkworm before each instar changes and previous to the cocoon production, which fixes the cocoon to a suitable substrate [32].

Sinohara and Asano [44] isolated glycopeptides from the proteolytic digestion of sericin, indicating the presence of glucosamine, galactosamine, mannose, and galactose. In 1979, Sinohara [45] found that* B. mori* sericin contains two types of oligosaccharide units. One consists of several mannose residues and two N-acetylglucosamine residues, one of which is linked to asparagine residue of protein core. The other oligosaccharide unit consists of an isolated N-acetylgalactosamine or a disaccharide *β*-galactosyl (1→3)-N-acetylgalactosamine, linked to the protein core in the serine or threonine residue.

### 3.2. Obtainment of Sericin

Removal of sericin gum from crude silk is based entirely upon the solubility in water [24]. The protein is typically obtained from the cocoon of* B. mori* but can also be extracted from the silk gland. In the traditional process of degumming in silk manufacturing, there is a common use of soaps and detergents [46], but this method can cause partial hydrolysis of sericin, reducing its natural weight and losing some functional properties [47].

With the objective of extracting and utilizing the sericin from cocoon of* B. mori*, four methods can be listed: high temperature, associated or not with high pressure by autoclaving; acidic, mainly citric acid solution; alkali, with sodium carbonate solution; and urea [24, 38]. All these methods can be modified in the relationship of temperature, time, chemical additive that was utilized, concentration of the solution, and others.

Kodama [48] reported that heating at high temperatures (100 to 105°C), used during the extraction in autoclaving, causes some change in the sericin molecule. The author also showed that sericin is soluble in water and has an isoelectric point slightly more acidic. Aramwit et al. [46] add that degumming by heat or heat under pressure has as advantage because it results in no impurity. The preparation conditions including temperature, pressure, and heating duration can largely mediate the molecular weight of sericin when it is extracted from cocoons. Namely, the molecular weight of sericin can be controlled by the extraction conditions [49].

Takasu et al. [41] use a saturated aqueous lithium thiocyanate containing 2-mercaptoethanol solution with ethanol precipitation for sericin extraction and show that sericin is composed of three major polypeptides, with molecular weight of 400, 250, and 150 kDa estimated by SDS-PAGE, which corresponds to sericins M, A, and P, respectively.

Kurioka et al. [50] tested the sericin extraction with an acidic solution using 1.25% citric acid, comparing the morphological and biochemical properties of the protein to that obtained with an alkali-degraded (0.5% sodium carbonate) and high temperature and pressure (autoclaving at 110, 115, and 121°C). The three extraction methods are equivalent to the yield of sericin powder. Morphologically, observing SEM (scanning electron microscope) images, in the acid degumming can be observed thin films with a leaf-like structure, in smaller size than compared to the alkali- and heat-degumming. Extraction with heat at 110°C has shown a trypsin inhibitor activity by 31%, 60% lower than the result of acid extraction. The alkali-degumming has no effect on the inhibition of trypsin. The trypsin inhibitor activity decreased 60% and 75% with increasing temperature to 115°C and 121°C, respectively.

Aramwit et al. [51] investigated the chemical properties and antityrosinase activities of sericin extracted from various methods. For extraction with high temperature and high pressure, cocoons of* B. mori* were autoclaved at 120°C and 15 lbf/in^2^ for 60 min. For the preparation by acid- and alkali-degraded was added to the cocoons 1.25% citric acid solution or 0.5% sodium carbonate solution, respectively, and boiled for 30 min. Cocoons were soaked into aqueous 8 M urea solution for 30 min and then refluxed at 85°C for 30 min for degumming with urea solution. The authors concluded that the sericin extracted with high temperature and pressure and by urea solution had higher yields compared with the other methods. Moreover, the sericin extracted by urea solution appeared to give the most clearly distinguishable protein bands in SDS-PAGE. When obtained with high temperature and high pressure, the sericin shows an endothermic degradation at 220°C, higher temperature than those obtained from other methods (210°C), implying that the use of chemicals during the extraction process influences the thermal stability of sericin. Other tests results revealed that the extraction process of sericin could affect the chemical structure of protein and change the amino acid composition. Among the four methods used in this study, extraction with urea had the most significant impact on sericin conformation. Still, regarding the inhibition of tyrosinase, urea-extracted sericin showed the highest antityrosinase activity, while the alkali-degumming has a minor.

Enzyme extraction is also used by other authors [52–55]. The use of enzyme to extract sericin from cocoon can be attributed to the discovery of cocoonase, a group of proteinases, which can attack the sericin bindings. The enzymes trypsin, papain, and bacterial enzymes were the main types used for the degumming process [56]. Trypsin, a proteolytic enzyme, hydrolyses the peptide bond between the carboxyl group of lysine or of arginine and amino groups of adjacent amino acids. Since sericin has a relatively high lysine and arginine content, it is easily hydrolysed by trypsin [57]. Papain exhibits a wide specificity in its action towards polypeptides and is an effective cocoon degumming enzyme [58]. Alkalase, a bacterial enzyme, and many other fungal protease enzymes have been standardized and found to be economically viable without chemical hazards [56].

The diverse methods of extraction of sericin, its origin, and the variety of cocoon, due to the different strains of* B. mori*, provide different sizes for the protein, which can be seen by the change in amino acid composition (Table 1) and molecular weight (Table 2), which can reflected in biological properties [59].

## 4. Sericin Properties and Biomedical Applications

The physicochemical properties and molecular heterogeneity of sericin influence on their functionality and these characteristics are directly influenced by extraction methods. Studies of biocompatibility and antioxidant potential, both* in vitro* and* in vivo*, have demonstrated that sericin is immunologically inert and have proven the safety and open wide possibility of applications of sericin in biomedicine, such as the food and cosmetic industries, supplement in the culture media, cryopreservation, wound healing, antitumour effect, various metabolic effects in organic systems, and indicate your use in tissue engineering and as a vehicle for drug delivery [26, 98].

### 4.1. Immunological Response

Silk fibres have been used in the biomedical field as sutures, since their biocompatible characteristics make them a promising biomaterial [99]. A few studies show the immune system activation front the silk proteins [100–102] and, historically, the hypersensitivity reactions were attributed to the sericin [4].

However, subsequent studies have shown a differential immunological role for sericin. An interesting finding regarding the immunological responses to silk has been presented by Panilaitis et al. [93] that examined the inflammatory potential of intact silk fibres and their* in vitro* extracts. The authors found that silk fibres and soluble sericin are immunologically inert in culture of murine macrophage cells, while insoluble fibroin particles can induce significant release of TNF-*α* (tumour necrosis factor-*α*). Even though sericin does not activate the immune system itself, it was found that when it covers the fibroin fibres, there is a strong macrophage in response to bacterial lipopolysaccharide. Thus, the authors confirm the low inflammatory potential of silk fibres, making them promising candidates for biomedical applications.

In a study involving cocoons of different lines of silkworms, Chlapanidas et al. [84] found that sericin has antiproliferative activities in peripheral blood mononuclear cells stimulated* in vitro*, as well as reducing the release of interferon gamma (IFN-*γ*), without having effects on the release of interleukin 10 (IL-10) and TNF-*α*, thus highlighting the applicability of sericin dermatologically, showing its anti-inflammatory role, related positively your biocompatibility, as well as protection against endogenous cells aggressions [84].

Sericin has been successfully added to the culture media of several cell lines and it does not promote any cytotoxicity, which indicates the safety of sericin to cells [65, 70].

Aramwit et al. [9] investigated the inflammatory mediators induced by sericin* in vitro* and* in vivo*. When the sericin was added to the culture media of mouse monocyte and alveolar macrophage cell lines, there was an increase in cell proliferation and the generation of TNF-*α* and interleukin-1 beta (IL-1*β*); however, this increase in cytokines does not activate other inflammatory cascades. In their* in vivo* assay, the authors used an 8% sericin cream, which was applied topically on wounds on the back of rats. After 7 days of treatment, there was a reduction of the expression of TNF-*α* and IL-1*β* in tissue and the overall wound healing was accelerated in treated animals. In this way, sericin promoted wound healing without exacerbating the inflammatory process.

Considering that a protein or new substance can be used as a biomaterial or a biomedical product, the immunological response is normally evaluated as an inflammatory reaction, with the expression or release of cytokines, especially IL-1*β* and TNF-*α*, both* in vitro* and* in vivo* [103]. Therefore, the sericin can be considered as a biocompatible protein, since it presents very low immunogenicity [98], and it can be utilized in various biomedical areas, as listed in sequence.

### 4.2. Antioxidant

Dietary antioxidants have been of great interest, especially due to the findings on the effect of free radicals in the body, which can have serious consequences if their products are not neutralized by an efficient antioxidant system [104]. In this sense, studies have shown the antioxidant properties of sericin* B. mori*. Kato et al. [10] showed, for the first time in* in vitro* study, that sericin inhibits lipid peroxidation in rat brain homogenate. The lipid peroxides, derived from polyunsaturated fatty acids, are unstable and may decompose into malondialdehyde [105, 106]; whose levels are associated with cardiovascular risk factors, hypertension, diabetes, and hyperlipidaemia [107]. Similarly, sericin was effective in inhibiting tyrosinase, the enzyme responsible for browning reactions of various foods and the synthesis of melanin, in addition to its role in cancer and neurodegenerative diseases [108], highlighting the interest in the study of substances with antityrosinase activity.

Cocoons of* B. mori* can provide natural pigments typically flavonoids and carotenoids that accumulate in sericin layers [109]. These pigments are known for their biological properties as antioxidants and antityrosinase. Aramwit et al. [51] demonstrated that the antityrosinase activity of sericin was greater when obtained from cocoons with pigments, but the potential was also present in cocoons submitted to the pigment extraction process, showing that sericin itself has a significant antityrosinase activity. Thus, sericin and pigment are responsible for antioxidant properties. Furthermore, the authors point out that the lineage of* B. mori* and the method of extraction of sericin affect their physical and chemical properties, influencing the antioxidant activity. Sericin extracted by urea solution, with molecular weight ranging from 10 to 225 kDa, has had the highest degree of antityrosinase activity, whereas alkali-degraded sericin showed no inhibition of tyrosinase. The high amount of arginine and valine amino acids, which can be observed in extraction by urea solution, is perhaps responsible for the antityrosinase activity, since arginine-containing peptides are the most tyrosinase-binding in shorter peptides and valine-containing peptides are the highest tyrosinase-inhibitor. Chlapanidas et al. [84] working with cocoons of 20 lineages of* B. mori* also demonstrated its influence on the antioxidant properties (antityrosinase, antielastase, and elimination of reactive oxygen species) of sericin.

Dash et al. [110] analysed the antioxidant and photo protector potential of sericin from* Antheraea mylitta*, against ultraviolet light B (UVB) in irradiated human keratinocytes. The analysis in flow cytometry revealed that previous treatment with sericin inhibited apoptosis induced by UVB, by inhibiting the expression of proapoptotic protein bax and upregulation of bcl-2, and it prevents the activation of caspase-3. There was also the inhibition of hydrogen peroxide formation in keratinocytes treated with UVB, indicating a role of sericin in preventing mitochondrial damage. In addition to these effects, intracellular reactive oxygen species (ROS) and activation of poly-ADP-ribose polymerase enzyme (PARP) were also decreased, directly involved in DNA cleavage processes, their own apoptotic process. The authors conclude that sericin, for these purposes, is a potent antioxidant and antiapoptotic agent. Likewise, the sericin antioxidant potential, extracted from* A. mylitta* cocoon, was demonstrated in skin fibroblasts (cell line AH927) exposed to hydrogen peroxide for 24 hours, using catalase, lactate dehydrogenase, and malondialdehyde as indicators [111].

In Takechi's et al. [112] study, the methods of 1,1-diphenyl-2-picrylhydrazyl (DPPH), chemiluminescence, and oxygen radical absorbance capacity (ORAC) proved a major antioxidant potential of sericin obtained from the yellow-green cocoon. According to the authors, the flavonoid pigment present in the sericin layers is responsible for this characteristic. By contrast, the electron spin resonance (ESR) shows that white sericin is better probably because the antioxidant potential of flavonoids pigments may not be involved in the elimination of hydroxyl radicals, detected by this method. Therefore, the results confirmed that all sericins have high antioxidant property against various free radicals, and the antioxidant property of the bread was improved by the addition of sericin powder.

Li et al. [66, 72] observed a protective effect of sericin in hepatic and gastric injuries caused by alcohol in mice. The treated animals showed higher alcohol elimination in urine, and this lowers concentration in serum. The sericin restored the normal parameters of antioxidant enzymes, demonstrating its protective role against lipid peroxidation and generation of ROS in the liver. Further, sericin recovered cell morphology and preserved the mitochondria integrity in gastric mucosa, probably based on its antioxidant potential.

The sericin significantly reduces intracellular ROS detected by fluorescence. Micheal and Subramanyam [113] suggested that the main constituent amino acids of sericin protect the midgut epithelial cells of* B. mori* and haemocytes from oxidative damage, probably by the ability of sericin to eliminate ROS.

The antioxidant properties of sericin could be related to your high serine and threonine content, whose hydroxyl groups' act chelating trace elements such as copper and iron [10]. Devi et al. [114], in their study about sericin from* Antheraea assamensis,* and Prasong [115], that compared the silk of* Samia ricini* with* B. mori*, concluded that the presence of polyphenols and flavonoids in sericin is responsible for its antioxidant role. Therefore, these studies suggest the use of sericin as a natural and safe ingredient for food and cosmetics industries.

### 4.3. Cosmetology

The use of sericin in cosmetic formulation, such as creams and shampoos, leads to an increase in hydration, elasticity, cleaning with less irritation, and antiaging and antiwrinkle effects [116–118] and also prevents nails from chapping and brittleness [119]. These applications are especially due to the presence of amino acids with hydrophilic side groups (80%), such as serine (30 to 33%), which has large capacity to absorb water. The sericin may also form a soft and smooth film on the surface of the skin, preventing the loss of water [120].

Padamwar et al. [121] studied* in vivo* moisturizing effect of sericin on human skin and found its action to decrease the impedance and increase in the level of hydroxyproline and hydration of the epidermal cells. The increase in hydration was attributed to the occlusive effect of sericin, which prevented the transepidermal water loss, responsible for skin dryness. The moisturizing power remained flat epidermal topography. The authors still argue that sericin has similar amino acid structure to filaggrin, present in the stratum corneum of the skin and acts in the natural hydration of the skin; and, thus, sericin itself is an important moisturizing agent.

The dryness is characteristic of a number of skin diseases such as atopic dermatitis and ictiosis, which lead to a decrease in free amino acids in the stratum corneum. Using silk proteins, fibroin and sericin, as treatment in an animal model of atopic dermatitis, Kim et al. [64] observed that adding 1% of sericin to the diet for 10 weeks caused an improvement in epidermal hydration. The consumption of sericin provided the increase of total filaggrin and free amino acids, as well increased of peroxisome proliferator-activated receptor (PPAR*γ*), peptidylarginine deiminase-3 (PAD3), and caspase-14, molecules responsible for the increase in expression of profilaggrin and filaggrin degradation of free amino acids, that recover dry skin conditions. Therefore, sericin is presented as a potential alternative therapy as an adjunct in the treatment of dry skin conditions such as atopic dermatitis.

### 4.4. Supplement in Culture Media and Cryopreservation

Studies in cell culture are often the first way to test new discoveries or technologies, particularly in research on cell therapy and regenerative medicine [70]. Many cell lines require culture media to remain viable; currently, the most widely used media are BSA (bovine serum albumin), which may possibly be contaminated by viruses, such as bovine spongiform encephalitis [65]. Furthermore, the cryopreservation of cell lines is the focus to the development of research into tissue engineering, and the media of BSA supplemented with 10% DMSO (dimethyl sulfoxide) are commonly used [122]. Considering that serum is the highest cost in cell culture [70], the development of new ways of supplementation and cryopreservation, especially those serum-free, is an important alternative.

Sericin obtained from the cocoon was added alone or combined with BSA in the culture media of mammalian cell lines (murine hybridomas 2E3-0, human hepatoblastoma HepG2, human epithelial HeLa cells, and human embryonal kidney 293 cells). The use of sericin with molecular weights between 6 and more than 67 kDa, known as small-sericin, increased cell proliferation in four lines, with positive results in concentrations from 0.01% to 0.1%, while higher concentrations (1%) were potentially dangerous. The sericin promoted an increase in cell viability, which became more pronounced when added to the BSA. Furthermore, the activity of the sericin did not change after autoclaving, showing its use as a supplement in culture media to stimulate cell proliferation [65].

From the study described above, Terada et al. [70] used the sericin with different molecular weights in cell culture. The sericin with higher molecular weight, 50 to 200 kDa, also stimulates cell proliferation, but it had not reached the sericin results of lower molecular weight which, in turn, increased the proliferation of hybridoma cells in various serum-free media, showing that the mitogenic effect is independent from the culture media.

Sasaki et al. [122] developed a serum-free freezing medium and, therefore, used 1% sericin of 30 kDa extracted from cocoons of* B. mori* together with PBS (phosphate buffered saline), 0.5% maltose, 0.3% proline, 0.3% glutamine, and 10% DMSO. These media showed the same cryoprotectant potential in P3U1 myeloma and Chinese-hamster ovary cells as the conventional medium of BSA containing 10% DMSO and were superior to all three commercial media. The media containing sericin also proved effective in cryopreservation of human, rat, and insect cell lines. Thus, the authors concluded that the use of supplemented media with small-sericin could act as universal media for cryopreservation of mammalian cells and insects.

Morikawa et al. [123] used sericin or FBS (fetal bovine serum) as medium for rat islets culture and there were no observed significant differences in survival and insulin secretion for 14 days. Ogawa et al. [83] tested the effect of sericin and FBS on islet cell survival and insulin production and, compared to Morikawa et al. [123], on the 12th day, all islets were morphologically intact in FBS culture, while only 50% were intact in sericin culture. On insulin production, sericin was effective in maintenance, but its effects were inferior to FBS.

Considering its antioxidant potential and the ability to eliminate free radicals, Kumar et al. [124] used different sericin concentrations (0.25, 0.5, 1.5, and 2%) and tested their cryoprotective action on buffalo spermatozoa. The supplementation of sericin in 0.25, 0.5, and 1% increased the spermatic motility, whereas concentrations of 0.25 and 0.5% were effective in preserving the integrity of the plasma membrane of spermatozoa and an increase in SOD activity (superoxide dismutase), an antioxidant enzyme, as well as reducing the concentration of malondialdehyde, the end product of lipid peroxidation. Thus, supplementation of low doses of sericin, 0.25 and 0.5%, is an effective cryoprotectant, which maintains the quality of semen and prevents oxidative stress.

Verdanova et al. [125] used sericin to develop serum-free media as well as to replace the medium cryoprotectant DMSO in human mesenchymal stromal cells (hMSCs) and immortalized human osteoblasts (SAOS-2 cell line). The supplementation of 1% sericin in 10% DMSO, as BSA substituent, proved to be efficient cryoprotection media for hMSCs cells, since they maintained their cell viability and ability to form colonies, which was not observed in SAOS-2 cells. When the sericin was used as the substituent of DMSO, the results showed that the reduction of DMSO is directly related to shorter survival of the cell lines studied, mainly in SAOS-2 cells. The authors conclude that sericin can be used as the substituent BSA, thus playing a role in preserving less mature lines, undifferentiated as hMSCs cells, while it does not act in this manner compared to differentiated cells, such as SAOS-2. Further, sericin was not efficient in replacement of DMSO in any of the studied lines.

### 4.5. Wound Healing

Several studies provide evidence of the healing properties of sericin, since it operates in stimulating the migration, proliferation, and production of collagen [126–128]. Aramwit et al. [127] discuss the importance of amino acid methionine of sericin in collagen synthesis, essential in the healing process. Tsubouchi et al. [129] examined human fibroblasts cultured in culture media containing sericin (400 kDa, known as sericin M) and observed cell growth of 250% in 72 hours, due to ease in connection of cell and media, which is dependent on repeated domains rich in serine found in the sericin. Tsubouchi et al. [129] added that this activity is dependent on the molecular weight and amino acid content of sericin.

*In vitro* test in model of injury (injury in monolayer cell with measurement of the expansion of cell population on surface) revealed that treatment with sericin (100 *μ*g/mL) induced migration of fibroblasts L929 and the wound closure time was significantly less than control [74]. In the wound, there is a loss of cell contact and the production of growth factors and cytokines occurs, initiating the proliferation and migration of cells, such as keratinocytes and fibroblasts [130]. Thus, the migration of fibroblasts is a crucial step towards healing, because it involves the proliferation, contraction, and collagen production [131], and sericin increases the population of fibroblasts into the injured area due to cell migration and/or proliferation [74].

In a clinical study, Aramwit et al. [74] used the standard antibiotic cream (silver sulfadiazine) with 8% sericin for the treatment of open wounds resulting from second-degree burns. The 29 patients had their burns treated with topical application of sericin, and blind evaluation showed that sericin accelerated wounds closure; the average time required to reach 70% of epithelialization of the burn surface to complete healing in treatment group was significantly shorter than control (without sericin), about 5–7 days. There was also a decrease in length of hospitalization and patients' pain, improving their quality of life. Aramwit et al. [9] also investigate the use of an 8% sericin cream on wounds on the back of rats and observed a reduction of tissue inflammatory cytokines and the overall wound healing was accelerated in treated animals. These results corroborate the morphological studies where sericin can activate collagen production [127, 128] and promote wound healing in rats [126, 132].

Sericin, with molecular weight ranging within 50–150 kDa, extracted from* B. mori* cocoons, was also used to form an 8% sericin cream for topical application in the treatment of uremic pruritus in patients with end-stage renal disease, demonstrating a greater skin hydration and less irritation and skin pigmentation. These clinical changes led to lower sensation of pain reported by patients using the Visual Analogue Scale and, consequently, improved quality of life in treated patients [39].

Nagai et al. [133, 134] and Nagai and Ito [135] used the small-sericin (30 kDa) as a treatment for corneal lesion in an animal model of type 2 diabetes mellitus. In their studies, the authors showed that instillation of sericin diluted in saline on corneal damage accelerated the healing process. Further, sericin was added to the culture media of an epithelial cell line of human cornea and was effective in increasing the cell proliferation and adhesion.

Besides injuries to the cornea, diabetes mellitus can cause degeneration in other tissues, especially on the central and peripheral nervous system. Song et al. [76] used a dose of sericin (2.4 g/kg) given orally for 35 days in an animal model of diabetes mellitus. The treatment reduced blood glucose and increased the expression of neurofilament protein in the sciatic nerve and the expression of nerve growth factor in L4–L6 spinal ganglion and anterior horn cells. However, the expression of neuropeptide Y in spinal ganglion and anterior horn cells significantly decreased. These results indicate that sericin protects the sciatic nerve and the nerve cells related against injuries caused by diabetes mellitus. Chen et al. [136] conducted a methodologically similar study and found that sericin also improves disorders of the growth hormone/insulin-like growth factor I axis caused by diabetes mellitus.

### 4.6. Antitumour Effect

The chemotherapy used in cancer treatment presents high cytotoxicity, affecting both normal cells and neoplastic, which limits its clinical applications [137]. In addition, another problem is the resistance to chemotherapeutic agents [138], making it necessary to search antitumour agents with lower toxicity and biocompatible, such as sericin.

Zhaorigetu et al. [62] studied the effect of 30% sericin supplementation in the diet of an animal model of colon tumorigenesis. Consumption of sericin for 115 days did not affect body weight and food consumption; however, there was a reduction in the incidence of colon adenoma. The antitumour effect of sericin results in lower cell proliferation rate, decrease in oncogenes expression, and reduction of oxidative stress. Sasaki et al. [77] found similar effects with the sericin supplementation also in a colon tumour model. In this study, supplementation of 3% of sericin for 5 weeks reduced the number of aberrant crypt foci, revealing its antitumour effect.

In order to clarify the mechanism involved in the antitumour action of sericin in colon cancer, Zhaorigetu et al. [78] supplemented the diet of an animal model of colon tumour with 3% of sericin for 28 days. In these animals, the use of sericin reduced the number of intestinal aberrant crypt foci and lipid peroxidation in colonic mucosa in 36% and 34%, respectively. The authors also observed that the sericin is not digested and, therefore, the strong antioxidant potential of sericin undigested present in the colon induces lower oxidative stress and tumorigenesis in the organ.

Kaewkorn et al. [73] studied the effect of sericin in the proliferation and apoptosis of colon tumour cells. The small-size sericin, 61–132 kDa, showed an inhibitory effect on human colorectal cancer cells (SW480) when compared to normal human fetal colonic mucosal cells (FHC). Further, sericin caused a reduction in cell viability by inducing apoptosis of tumour cells with increased activity of caspase-3 and reduction of Bcl-2 expression, an antiapoptotic protein. The sericin did not induce apoptosis in control cells, acting as chemoprotector against colon cancer cells.

Sericin was used for Zhaorigetu et al. [82] to test its protective effects in an animal model of skin tumours induced by chemical substances, 12-O-tetradecanoylphorbol-13-acetate (TPA, oxidative stress inducer) and 7,12-dimethylbenz(*α*)anthracene (DMBA). Topical application of 2.5 mg dose led to a delay in tumour appearance in 1 week when compared with untreated group with sericin, whereas the dose of 5 mg of sericin inhibited its appearance for 15 weeks, and a small tumour was observed at the end of the experiment at the 16th week. In addition to the inhibition of tumorigenesis, sericin led to a reduction in the number (cell proliferation) of skin tumours in mice. There was a marked decrease in inflammatory response, by a decrease in leukocyte infiltration by TPA, and epidermal hyperplasia. Topical application of sericin, prior to TPA, also reduced the expression of epidermal protooncogenes, c-fos and c-myc, and proinflammatory mediator cyclooxygenase-2 (COX-2). Thus, the chemoprotector effect of sericin of mouse skin tumour occurred for its action in reducing the oxidative stress, inflammatory response and TNF-*α*.

### 4.7. Metabolic Effects

#### 4.7.1. In Gastrointestinal Tract

Functional gastrointestinal disorders are common clinical conditions, characterized by the presence of symptoms in the absence of organic, structural, or metabolic abnormalities [139]. Considering its antioxidant potential and its hydrophilic characteristic, the use of sericin in the gastrointestinal tract has been investigated. Sasaki et al. [61] supplemented the diet of rats with 3% of sericin for 12 days. The use of sericin caused an increased absorption of zinc (41%), iron (41%), magnesium (21%), and calcium ions (17%), improving the bioavailability of these elements; however, it does not change the serum concentration and urinary excretion of these elements analysed by the atomic absorption spectrophotometer. Despite its resistance to proteases [81], undigested sericin seems to increase the solubilisation of ions in the gastrointestinal tract by ion chelation with its hydroxyl and carboxyl groups, leading to increased availability and indicating potential use of sericin as a functional food [61].

Sasaki et al. [81] tested the sericin as a supplement in an animal model of constipation. In this study, the addition of 4% of sericin in the diet for 14 days did not alter body weight or food consumption but increased faecal water content, preventing constipation caused by atropine, agent that causes a reduction of acetylcholine release by the parasympathetic nervous system [140]. The ability of sericin to retain water as well as other unfermented fibres [141] may be indicated as being responsible for its role in the improvement of constipation. Still, sericin raised the dry faecal weight and the nitrogen content in faeces, indicating a lower protein digestion. The pattern of amino acids in the faeces was similar to the amino acid content of sericin and the apparent low digestibility of sericin was confirmed by the authors in an* in vitro* assay, since this was not digested by pepsin and pancreatin, indicating a potential for resistance to proteases related probably to its high serine content [81].

Okazaki et al. [79] supplemented the diet of rats with 40 g/kg of sericin for 3 weeks to assess its effect on the intestinal lumen of mice fed with a high-fat diet. In this study, sericin increased the amount of immunoglobulin A (IgA) faecal in the colon, associated with lower risk of colon cancer and ulcerative colitis [142], and increased the wet weight of cecal digesta and presence of faecal mucins, especially acetate, which can be related to the reduction of low-density lipoproteins. The consumption of sericin did not alter the microflora and secondary bile acids, although it reduced faecal cholic acid content (primary bile acid). Accordingly, sericin can be considered a prebiotic, as it promotes colon health by modulating the immune response and the intestinal barrier function.

#### 4.7.2. In the Circulatory and Immune Systems

Bioactive peptides derived from dietary proteins are important in modulating physiological functions [143], and Onsa-ard et al. [55] demonstrated that sericin, particularly oligopeptides with less than 5 kDa, has hypotensive potential, promoting vasodilation and relaxation of smooth muscle of the artery wall, whose temporary effect is dose-dependent. The authors believe that the oligopeptides of the sericin can have an antagonistic action on calcium channels by blocking them, leading to muscle relaxation. Another mechanism involved is agonist interaction of oligopeptide with nitric oxide and prostacyclin, activating the way that promotes smooth muscle relaxation. Thus, evidence supports the indication of sericin as a vascular modulator.

The anticoagulant potential of sericin was investigated by Tamada et al. [144]. The authors sulphated the serine residue of sericin protein extracted from cocoons of* B. mori* using chlorosulfonic acid. They found that higher molecular fractions had higher anticoagulant activity, estimated to be 1/10 to 1/20 of heparin. Sano et al. [145] investigated as sulphated sericin might be involved with the coagulation cascade mechanisms to elucidate its anticoagulant mechanisms. The authors found that sulphated sericin interferes with fibril accumulation of fibrin, without delaying the initial polymerization process.

Considering the regulatory role of dietary protein in the immune system [146], Keawkorn et al. [68] investigated the effect of sericin addition (4%) on mice diet for 20 weeks. In this study, the consumption of sericin did not alter body weight, food intake, and blood cells count but reduced the percentage of CD8a and CD80 cells. CD8a is a surface marker of cytotoxic T cells and natural killer (NK), responsible for immune response in the elimination of tumour cells and viral infections [147]; and CD80 is related to antitumour immunity mediated by T cells [148]. The mechanism underlying the reduced CD8a and CD80 cells is unknown but is presumably related to amino acidic content of sericin.

The effects of oligopeptide with molecular mass < 5 kDa derived from sericin on NK cells activity was verified by Jantaruk et al. [149].* In vitro* exposure of peripheral blood mononuclear cells to oligopeptide derived from sericin resulted in increased NK activity against K562 target cells, with a dose-dependent effect. The authors found that the effect on NK cells is indirect, due to induction of interleukin-2 (IL-2) and IFN-*γ*.* In vivo*, oral administration of oligopeptides derived from sericin in mice, also caused increased activity of NK cells and an increase in IL-2 concentrations. Thus, it is evident the possibility of application of oligopeptides derived from sericin in the treatment of tumours and infectious diseases.

#### 4.7.3. On Lipid Metabolism and Obesity

Obesity, a worldwide epidemic, is characterized by an excessive body fat increase, accompanied by a number of comorbidities [150]. Considering the difficulty in combining physical activity with improved eating habits [151], researches with potential therapeutic agents for obesity are gaining prominence [152]. In this regard, various studies point to the promising effects of sericin on lipid metabolism and obesity.

Okazaki et al. [63] examined the effect of sericin on lipid and carbohydrate metabolism in mice fed with a high-fat diet. The addition of 4% of sericin on the diet for 5 weeks did not alter body weight, food consumption, and fat weight but reduced serum concentrations of cholesterol, triglycerides, free fatty acids, phospholipids, very low-density lipoproteins (VLDL), and low-density lipoprotein (LDL). Sericin also decreased triglycerides and lipogenic enzymes in the liver, increased the serum adiponectin (64%), and improved glucose tolerance. The authors suggest that the high serine content associated with low concentrations of methionine may be appointed as responsible for the hypolipidemic action of sericin. According to Verhoef et al. [153], serine supplementation attenuates the plasma homocysteine concentration induced by dietary methionine, and homocysteine is known to promote insulin resistance [154]. Thus, supplementation of sericin has a beneficial role in the metabolic syndrome conditions resulting from a high-fat diet.

Limpeanchob et al. [67] reported a reduction of non-HDL (high-density lipoprotein) cholesterol and total cholesterol in mice that received an oral solution of 10, 100, or 1000 mg/kg of sericin for 14 days and no changes in triglycerides and HLD levels. The authors found that sericin reduces cholesterol absorption in the intestine in an* in vitro* model with differentiated Caco-2 cells, thus resulting in reduction of plasmatic cholesterol, probably due to the effect of sericin in inhibition of cholesterol absorption and its solubilisation into micelles. According to Morita et al. [155], there is a correlation between low methionine content, as shown by the sericin, and lowering cholesterol. Furthermore, the low digestibility of sericin [81] may influence the bowel function throughout its length, blocking the intestinal absorption of cholesterol.

Ali and Arumugam Sarasa [80] used a crude extract of* B. mori* for 45 days in hyperlipidemia and atherosclerotic mice. The treatment improved the lipid profile and reduced the extent of atherosclerotic lesions, which, according to the authors, is due to sericin, who acted beneficially in the clinical condition studied, probably by its amino acid content.

Seo et al. [156] investigated the effects of silk proteins supplementation (2%) of mice on high-fat diet for 6 weeks. The treated group with the highest proportion of sericin (50%) showed beneficial effects in combating hyperlipidemia and obesity, as reduced weight gain and weight of fats, and increased expression of enzymes involved in *β*-oxidation with concomitant reduction of lipogenic enzymes. Still, there was decrease of serum leptin, resistin, and TNF-*α*, adipokines related to inflammatory profile of obesity [157] and elevation in adiponectin, with antiatherosclerotic, anti-inflammatory, and hepatoprotective function [158]. According to the authors, the overall improvement in plasma lipid profile and increase of cholesterol and triglyceride levels in stools are due to the low digestibility of sericin [81], which can act as a dietary fibre and accelerate faecal lipid excretion, reducing their plasmatic concentration.

### 4.8. Tissue Engineering: Epithelial and Connective Tissue Repair

The tissue engineering normally uses biomaterials that are a suitable scaffold that possess the specific structure of the tissue it replaces and must be capable in turn of being replaced in time via the ingress of new cells [159, 160].

Teramoto et al. [161] show that only the addition of 10% alcohol in sericin solution forms a hydrogel, without necessity of crosslinking by chemicals or irradiation, indicating the sericin hydrogel as a natural biomaterial. Although your potential, pure sericin forms fragile films and is difficult to use as biomaterial in tissue engineering [162] and different strategies have been applied to increase the physical properties of sericin [98].

Mandal et al. [162] fabricated a sericin/gelatin blended 2D films and 3D scaffolds using sericin from* A. mylitta* cocoon and glutaraldehyde as crosslinking agent. The sericin/gelatin combination structure possessed uniform pore distribution and homogeneous morphology, improved mechanical strength, and have high swellability. In* in vitro* test, at where feline fibroblast cells (AH 927) were cultured, there were attachment and proliferation of the cells on sericin/gelatin blended 2D films and 3D scaffolds, which became toxic at higher concentrations of sericin. Nayak et al. [163] also studied a sericin membrane from* A. mylitta* cocoon crosslinking with glutaraldehyde and observed increase in physical properties, associated with a slow enzymatic degradation and enhanced fibroblast cell attachment and viability. This result provides a perspective of sericin as biomaterial.

Nayak et al. [164] constructed 3D porous sericin matrices using genipin as crosslinked and used chitosan matrices as control, with the objective of developing an effective tissue-engineered skin replacement. Histological analysis indicates a multilayered stratified epidermal layer of keratinocytes, demonstrating that sericin matrices form epidermal and dermal components* in vitro*. Presence of involucrin, collagen IV, and fibroblast surface protein can be observed, associated with no significant amount of proinflammatory cytokines (TNF-*α*, IL-1*β*, and nitric oxide) production in macrophages grown on the sericin matrices. The biostability and good biocompatibility indicate 3D sericin matrices as skin equivalent tissue in wound repair.

A sericin/collagen membrane using glutaraldehyde as crosslinking was studied by Akturk et al. [165] and tested for your wound dressing potential. The membranes were not attacked by microorganisms and were biocompatible. Fibroblasts and keratinocytes attached the membranes, acquired your morphological characteristics, and proliferated. When implanted subcutaneously, the membrane was involved by a fibrous capsule and induced an acute inflammation before your complete degradation. The authors concluded that a sericin/collagen membrane had good mechanical and swelling properties, increased oxygen permeability and cellular attachment, and associated with antibacterial resistance, forming a potential alternative for wound dressing.

Siritientong et al. [166] tested the clinical potential of an ethyl alcohol-precipitated silk sericin/polyvinyl alcohol scaffold as a wound dressing, in comparison with Bactigras®, a commercially available wound dressing. The sericin biomaterial possesses appropriate properties and it can be applied with safety and high efficacy compared to Bactigras. The clinical evaluation revealed no evidence of skin irritation, demonstrated accelerated healing, and reduced pain compared with wounds treated with Bactigras.

Other researches also study the sericin as a biomaterial and its use in regenerative medicine, wound dressing, and tissue engineering [89, 167–170]. According to Lamboni et al. [98], the incorporation of sericin in skin repair and wound healing materials forms a potential biomaterial, whereby causing enhanced adhesion, migration, and growth of fibroblast and keratinocytes, increased collagen production, and promoted reepithelialization in skin wounds.

Pure sericin films form a low profile of mechanical properties that impedes your utilization as bone tissue engineering; however, its mitogenic capacity can promote bone synthesis and induce the nucleation of bone-like hydroxyapatite (HA) [98]. Zhang et al. [171] prepared a HA/sericin composite film by mineralization of a flexible ethanol-treated sericin film. The HA/sericin composite forms a 3D structure with poor crystallinity that present an excellent cell viability of human osteosarcoma MG-63 cells that grows in the direction of c-axis, similar to natural bone mineral.

Nayak et al. [172] investigated the functionalization of metallic implant titanium that was immobilized with silk protein sericin (*A. mylitta*) using glutaraldehyde as crosslinked. The authors show that the surface modification with sericin enhances the osseointegration and osteoconduction, proved by the growth and proliferation of mouse MG-63 osteoblasts, increase of alkaline phosphatase and osteocalcin, and absence of TNF-*α*, IL-1*β*, and nitric oxide production by RAW 264.7 murine macrophages and macrophage-osteoblast cells. Therefore, use of sericin is a promising strategy to influence the osseointegration of titanium implants and provides bone tissue engineering.

Dinescu et al. [173] tested a 3D collagen-sericin scaffold combined with 10% hyaluronic acid and 5% chondroitin sulphate, which formed a porous and homogeneous structure, similar to that of cartilage extracellular matrix. The scaffolds allowed a rapid swelling and motility of human adipose-derived stem cells, forming a potential biomaterial for cartilage tissue engineering. In another study, Dinescu et al. [174] used a 3D collagen-sericin scaffold and cultured human adipose-derived stem cells that stimulated the biocompatibility, with increased cell adhesion and proliferation, and provided PPAR*γ*2 overexpression, that upregulated expression of adipogenic markers, serving as a candidate for soft tissue reconstruction.

### 4.9. Vehicle for Drug Delivery

An optimal effect of the drug is achieved when its release profile is both reliable and controlled and, eventually, a delivery system that is compatible and presents an adjustable morphology is necessary for that to occur, as the silk proteins [175]. Sericin can be used for drug delivery due to its chemical reactivity that enable the easy binding of other molecules and pH responsiveness, allowing the fabrication of small materials [98].

Wang et al. [176] fabricated a covalently crosslinked 3D pure sericin gel and observed that it is becoming an injectable material, promotes cell adhesion and long-term survival, and possesses multiple physical and chemical properties that provides a sustained drug release ability, which can tailored and customized to different tissue repair applications, serving as a multifunctional platform for cell therapy. Zhang et al. [177] proved that sericin can improve the properties of alginate hydrogels and concluded that this association exhibits many advantages, in particular in mechanical property, degradation, photoluminescent property, and cell adhesion, besides supporting effective cell growth, long-term survival, and migration. This study indicates that the sericin/alginate hydrogels may serve as a versatile platform for delivering cells and drugs. The sericin may be used pure or conjugated with others polymers to produce matrices, particles, and hydrogels that improve the drug delivery capacity [98].

Nishida et al. [178] used different molecular sizes and concentrations of sericin to form film, gel, and sponge and tested your release properties of the charged protein fluorescein isothiocyanato-albumin. The major concentration of sericin determined an effective release of a charged drug protein, and the film form was the best releasing.* In vivo* test, using rats as model, shows that film and gel are encapsulated in collagen-like material and have their size and weight decreased with the time. The drug remained for approximately 3 weeks in gel form and for more 6 weeks in the sericin film, and none form induced inflammation upon the implantation. Sericin forms are suitable for use as a drug-releasing biomaterial.

Parisi et al. [179] used a sericin/poly(ethyl cyanoacrylate) nanosphere that was polymerized with fenofibrate, a lipophilic drug used as a lipid-regulating agent. The* in vitro* studies show an increase of 70% of absorbable amount of fenofibrate when incorporated into the synthesized nanoparticles, results that were confirmed* in vivo*, with an improved in plasmatic lipid levels and reduction in lipid accumulation in liver. So, sericin/poly(ethyl cyanoacrylate) nanospheres can be used as delivery system for poorly water-soluble drugs.

Zhang et al. [96] used silk sericin with molecular mass within 10–70 kDa and prepared sericin/insulin bioconjugates, obtained by crosslinking with glutaraldehyde. The half-life of sericin/insulin conjugates* in vitro* was 2.2 and 2.7 times more than bovine serum albumin/insulin conjugates and intact insulin, respectively, and your pharmacological activity in mice was over 4 times longer than that of the native insulin and not induced any antigenic response. The physicochemical and biological stability of insulin are improved with sericin conjugation.

Besides the use of sericin to form bioconjugates and act as a drug delivery system, many authors have formulated microspheres, nanospheres, or particles with other composts with objective of improving the releasing of their own sericin, which has, by itself, great properties in biomedical application. Aramwit et al. [87] fabricated chitosan/sericin microspheres at different composition and observed that the prepared at 50/50 release sericin in the most sustained behaviour, probably due to the strong ionic interaction of the composts. The microspheres at any concentrations were not toxic to L929 mouse fibroblast cells and were continuously degraded and remained around 20% after 14 days, suggesting the chitosan/sericin microspheres as a wound dressing material to achieve the sustained release of sericin. Khampieng et al. [86] conjugated silk sericin with alginate nanoparticles, and the release profile of sericin exhibited initial rapid release, consequently with sustained release, and confirmed the hypothesis that this gel can inhibit inflammation induced by carrageenan.

## 5. Conclusion

Silk protein sericin is a natural polymer produced and secreted by the silk gland insect* B. mori*. Sericin is a water-soluble glycoprotein and comprises 25 to 30% of the cocoon weight; it is characterized by the presence of 18 amino acids, with strong polar side groups (hydroxyl, carboxyl, and amino groups) and high content of serine, aspartic acid, and glycine, resulting in a hydrophilic protein. Sericin is splicing alternative product of genes* Ser1*,* Ser2*, and* Ser3*, which provides a high molecular heterogeneity, 20 to 400 kDa, and variation on amino acid molar percent. The physicochemical properties of sericin, which mainly depend on the method of sericin isolation and the lineages of the silkworm, affect its functional properties and make sericin a potential biocompatible material for biomedical applications.

## Figures and Tables

**Table 1:** Composition of amino acids of sericin^1^.

Amino acid	Yamada [60]	Kato et al. [10]	Sasaki et al. [61]; Zhaorigetu et al. [62]; Okazaki et al. [63]; Kim et al. [64]	Terada et al. [65]	Li et al. [66]	Limpeanchob et al. [67]	Aramwit et al. [39]; Aramwit et al. [51]	Keawkorn et al. [68]
Aspartic acid	18,46	17,8	19,1	18,0	19,18	16,70	15,64	16,7
Serine	28,58	31,0	30,4	32,2	28,89	33,40	33,63	33,4
Glutamic acid	4,84	4,4	4,1	4,6	6,98	4,40	4,61	4,4
Glycine	16,80	19,1	12,2	15,7	10,75	13,50	15,03	13,5
Histidine	0,94	1,0	0,9	1,3	3,95	1,30	1,06	1,3
Arginine	3,22	3,9	2,8	1,8	4,39	3,10	2,87	3,1
Threonine	9,92	8,0	6,0	8,4	7,81	9,70	8,16	9,7
Alanine	5,00	3,8	4,6	5,3	4,65	6,00	4,10	6,0
Proline	0,53	0,4	0,8	0,6	<0,05	0,70	0,54	0,7
Cysteine	0,53	<0,05	<0,05	<0,05	<0,05	0,20	0,44	0,2
Tyrosine	3,33	3,3	3,8	3,7	4,24	2,60	3,45	2,6
Valine	2,79	3,1	2,6	3,6	3,12	2,80	2,88	2,8
Methionine	0,10	<0,05	<0,05	<0,05	<0,05	0,04	3,39	0,04
Lysine	2,58	2,7	10,2	2,5	3,51	3,30	2,35	3,3
Isoleucine	0,63	0,4	1,4	0,7	0,83	0,70	0,56	0,7
Leucine	1,03	0,8	0,6	1,1	1,12	1,10	1,00	1,1
Phenylalanine	0,44	0,2	0,4	0,4	0,58	0,50	0,28	0,5
Tryptophan	—	—	—	—	—	0,70	—	0,2

^1^Values are presented in molar percent.

**Table 2:** Diverse methods of extraction of sericin of *B. mori*, molecular weight and its origin.

Methods	Molecular weight	Origin	Authors
Hot water at 80°C and 120°C 0,5% Na_2_CO_3_ aqueous solution at 80°C and 120°C	20–400 kDa <100 kDa	Cocoon	Gimenes et al. [69]
LiSCN saturated solution Silk gland fluid, shaken gently for 30 min	20–400 kDa	Cocoon Silk gland	Takasu et al. [41]
Autoclave at 120°C for 30 min Heated at 100°C for 10 min	12–66 kDa	Cocoon	Yang et al. [49]
0,2% Na_2_CO_3_ solution heated at 95°C for 120 min	6 and 67 kDa	Cocoon	Terada et al. [65]
Autoclave at 120°C for 30 min Heated at 95°C for 120 min	10–70 kDa	Cocoon	Terada et al. [70]
Distilled water and bromelain solution heated at 55°C for 60 min	10–250 kDa	Cocoon	Sonjui et al. [71]
Autoclave at 120°C for 60 min 1,25% citric acid solution heated for 30 min 0,2% Na_2_CO_3_ solution 8 M urea solution	20–220 kDa	Cocoon	Aramwit et al. [51]
Autoclave at 100°C and 105°C for 60 min	—	Cocoon	Kodama [48]
Autoclave	—	Cocoon	Li et al. [66]; Keawkorn et al. [68]; Li et al. [72]; Kaewkorn et al. [73]; Aramwit et al. [74]; Kitisin et al. [75]
Soaking, water decoction, filtration and condensation.	—	Cocoon	Song et al. [76]
Heated in deionized water at 95°C for 120 min	—	Cocoon	Kato et al. [10]; Sasaki et al. [61]; Sasaki et al. [77]; Zhaorigetu et al. [78]; Okazaki et al. [79]
Autoclave for 30 min, followed by enzymatic hydrolysis by protease	—	Cocoon	Onsa-ard et al. [55]
110°C for 300 min	65 kDa	Cocoon	Kim et al. [64]
1% NaCl solution	—	Cocoon	Ali and Arumugam Sarasa [80]
0,2% Na_2_CO_3_ solution heated at 95°C for 120 min	—	Cocoon	Zhaorigetu et al. [62]; Okazaki et al. [63]; Sasaki et al. [81]; Zhaorigetu et al. [82]; Ogawa et al. [83]
Autoclave at 121°C for 60 min	50–150 kDa	Cocoon	Aramwit et al. [39]
Autoclave at 120°C for 60 min	—	Cocoon	Aramwit et al. [9]; Chlapanidas et al. [84]; Saetae and Magaraphan [85]; Khampieng et al. [86]; Aramwit et al. [87]; Purwar et al. [88]; Siritientong and Aramwit [89]
Fluid at the anterior and the middle silk gland	20–400 kDa	Silk gland	Sprague [90]
Autoclave at 120°C for 40 min	20–400 kDa	Cocoon	Da Silva et al. [59]
Autoclave at 110°C for 480 min	—	Cocoon	Lee et al. [91]
0,02 M Na_2_CO_3_ solution heated for 45 or 60 min	—	Cocoon	Martínez-Mora et al. [92]; Panilaitis et al. [93]
Electrolytic alkaline water at 95°C for 7 or 13 hours	5–18 kDa	Cocoon	Ogino et al. [94]
Autoclave at 120°C for 60 min	200 kDa	Cocoon	Turbiani et al. [95]
Autoclave	14 and 97 kDa	Cocoon	Wu et al. [11]
Autoclave at 120°C for 60 min	10–200 kDa	Cocoon	Zhang et al. [96]
Autoclave at 120°C for 40 min	—	Cocoon	Da Silva el al. [97]
